# Effects of hindlimb unloading on the mevalonate and mechanistic target of rapamycin complex 1 signaling pathways in a fast‐twitch muscle in rats

**DOI:** 10.14814/phy2.15969

**Published:** 2024-03-07

**Authors:** Munehiro Uda, Toshinori Yoshihara, Noriko Ichinoseki‐Sekine, Takeshi Baba

**Affiliations:** ^1^ School of Nursing Hirosaki Gakuin University Hirosaki Aomori Japan; ^2^ Graduate School of Health and Sports Science Juntendo University Inzai Chiba Japan; ^3^ Faculty of Liberal Arts The Open University of Japan Chiba Japan; ^4^ School of Medicine Juntendo University Inzai Chiba Japan

**Keywords:** mitochondria, mTORC1, prenylation, skeletal muscle atrophy, small GTPase

## Abstract

Fast‐twitch muscles are less susceptible to disuse atrophy, activate the mechanistic target of rapamycin complex 1 (mTORC1) signaling pathway, and increase protein synthesis under prolonged muscle disuse conditions. However, the mechanism underlying prolonged muscle disuse‐induced mTORC1 signaling activation remains unclear. The mevalonate pathway activates the mTORC1 signaling pathway via the prenylation and activation of Ras homolog enriched in brain (Rheb). Therefore, we investigated the effects of hindlimb unloading (HU) for 14 days on the mevalonate and mTORC1 signaling pathways in the plantaris muscle, a fast‐twitch muscle, in adult male rats. Rats were divided into HU and control groups. The plantaris muscles of both groups were harvested after the treatment period, and the expression and phosphorylation levels of metabolic and intracellular signaling proteins were analyzed using Western blotting. We found that HU increased the expression of 3‐hydroxy‐3‐methylglutaryl‐coenzyme A reductase, the rate‐limiting enzyme of the mevalonate pathway, and activated the mTORC1 signaling pathway without activating AKT, an upstream activator of mTORC1. Furthermore, HU increased prenylated Rheb. Collectively, these findings suggest that the activated mevalonate pathway may be involved in the activation of the Rheb/mTORC1 signaling pathway without AKT activation in fast‐twitch muscles under prolonged disuse conditions.

## 1 INTRODUCTION

Prolonged skeletal muscle disuse induces muscle atrophy, although the magnitude of the atrophy varies among muscles (Baehr et al., 2017; Ciciliot et al., 2013; Thomason & Booth, 1990). Skeletal muscle atrophy of mice and rats in the hind limbs during unloading has been well‐established (Thomason & Booth, 1990), with fast‐twitch muscles, such as the plantaris muscle, exhibiting a lower degree of atrophy than the slow‐twitch soleus muscle (Baehr et al., 2017; Ciciliot et al., 2013; Thomason et al., 1987; Thomason & Booth, 1990). Thus, muscles that are less susceptible to disuse atrophy must possess as‐yet unknown protective mechanisms, the elucidation of which may help to prevent muscle atrophy.

Disuse‐induced skeletal muscle atrophy results from an imbalance between protein synthesis (downregulated) and protein degradation (upregulated) (Bodine, 2020). Unlike slow‐twitch muscles, fast‐twitch muscles exhibit a temporary decrease in protein synthesis after 3 days of hindlimb unloading (HU) but tend to recover subsequently, approaching control levels after 14 days (Baehr et al., 2017). Protein synthesis is activated by the AKT/mechanistic target of rapamycin complex (mTORC) 1 signaling pathway, in which activated AKT serves to activate mTORC1. In contrast, sustained activation of the mTORC1 signaling pathway activates a negative feedback loop to inhibit AKT and enhances the signaling pathways involved in protein degradation (Bentzinger et al., 2013; Kaiser et al., 2022; Tang et al., 2014). Thus, the mTORC1 signaling pathway participates in both protein synthesis and degradation. Prolonged muscle disuse does not activate the AKT/mTORC1 signaling pathway in slow‐twitch muscles (Castets et al., 2019; Hornberger et al., 2001; Tang et al., 2014). In contrast, prolonged immobilization and denervation can activate the mTORC1 signaling pathway in fast‐twitch muscles (Castets et al., 2019; Machida et al., 2012; Tang et al., 2014; You et al., 2015, 2021); however, the contribution of AKT to this mTORC1 activation remains unclear. Thus, the activation of mTORC1 in fast‐twitch muscles by prolonged muscle disuse may promote protein degradation through negative feedback depending on the time after muscle disuse, the degree of mTORC1 activation, and the model employed. However, the mTORC1 signaling pathway may be associated with enhanced protein synthesis and reduced susceptibility to muscle atrophy in fast‐twitch muscles following prolonged disuse.

The mTORC1 signaling pathway is activated by insulin‐like growth factor 1 (IGF‐1), catecholamine, and amino acid during muscle hypertrophy (Sartori et al., 2021). Conversely, growth/differentiation factor 8 (myostatin) was found to suppress this pathway (Sartori et al., 2021). However, the mechanism underlying muscle disuse‐induced mTORC1 signal activation remains unclear (Bodine, 2022). Fourteen days of HU was shown to decrease or not impact IGF‐1 content in fast‐twitch muscles of rats (Yimlamai et al., 2005). In addition, plasma catecholamine concentrations and β_2_‐adrenergic receptor expression in the muscles of rats were unaltered after 10 days of casted immobilization (Sato et al., 2011). Furthermore, myostatin was reportedly increased or unaltered by muscle disuse (Brooks & Myburgh, 2014), and mothers against decapentaplegic homolog 2 and 3, downstream signaling proteins of myostatin, were activated by immobilization‐induced muscle atrophy in mice (Tando et al., 2016). Therefore, extracellular signals are unlikely to activate mTORC1 signaling in unloaded muscles. A previous study has reported that muscle disuse‐mediated activation of the mTORC1 signaling pathway depends on increased intracellular amino acids induced by proteasome activation (Quy et al., 2013). However, muscle disuse activates the proteasome in the soleus muscle but not in the mTORC1 signaling pathway (Baehr et al., 2017; Bodine et al., 2001; Castets et al., 2019; Hornberger et al., 2001; Tang et al., 2014). Thus, factors other than amino acid metabolism may be involved in activating the mTORC1 signaling pathway in unloaded fast‐twitch muscles.

Another candidate potentially involved in the activation of the mTORC1 signaling pathway is the mevalonate pathway in intracellular metabolic processes. The mevalonate pathway is primarily supplied with substrates by mitochondria and involved in cholesterol biosynthesis and prenylation as a posttranslational protein modification (Mullen et al., 2016; Pietrocola et al., 2015). Treatment with statins, which inhibit the rate‐limiting enzyme 3‐hydroxy‐3‐methylglutaryl‐coenzyme A reductase (HMGCR) in the mevalonate pathway, downregulates the AKT signaling pathway and increases the muscle‐specific ubiquitin ligase MAFbx/atrogin‐1 (Hanai et al., 2007; Sanvee et al., 2021). In addition, the mevalonate pathway contributes to the mTORC1 signaling pathway activation through prenylation of the Ras homolog enriched in brain (Rheb) (Basso et al., 2005; Castro et al., 2003; Sanvee et al., 2021; Xu et al., 2015). However, the role of the mevalonate pathway in the AKT/mTORC1 signaling pathway in fast‐twitch muscles during muscle disuse warrants further investigation.

To clarify the mechanisms underlying the activation of the mTORC1 signaling pathway in fast‐twitch muscles during disuse, we investigated the effects of HU for 14 days on the mevalonate pathway, along with its upstream metabolic pathway and the AKT/mTORC1 signaling pathways, in the plantaris muscles of rats using Western blotting. Specifically, we evaluated the levels and activation of proteins related to the glycolytic pathway, which is upstream of mitochondria and is activated by muscle disuse (McMillin et al., 2021; Stein & Wade, 2005; Turinsky, 1987), mitochondrial rate‐limiting enzymes in the tricarboxylic acid (TCA) cycle supplying substrates to the mevalonate pathway (Mullen et al., 2016; Pietrocola et al., 2015) and enzymes that convert cytosolic citrate to mevalonate (Mullen et al., 2016; Pietrocola et al., 2015), and proteins involved in mTORC1 signaling activation, including AKT and Rheb. Our findings enhance the overall understanding of the association between metabolic processes and the AKT/mTORC1 signaling pathway in fast‐twitch muscles and provide insights into how these muscles avoid atrophy after prolonged disuse.

## 2 MATERIALS AND METHODS

### 2.1 Animals

All experiments were approved by the Institutional Animal Care and Use Committee of Juntendo University (approval number: H27‐05) and were conducted according to the guiding principles for the Care and Use of Laboratory Animals set by the Physiological Society of Japan. Almost all experimental methods used in this study are similar to those described in our previous report (Uda et al., 2020). Fifteen‐week‐old male Fischer F344/N rats (*n* = 12) were obtained from Japan SLC, Inc. (Shizuoka, Japan). The rats were housed under a 12:12‐h light–dark cycle in a controlled environment and provided with food and water ad libitum. One week after arrival from the vendor, the rats were randomly divided into the control (CON; *n* = 6) and HU (*n* = 6) groups.

### 2.2 Hindlimb unloading

Each rat in the HU group was exposed to tail suspension for 14 days using the method described by Yoshihara et al. (2015). Briefly, a tail cast was applied to each rat. The tail cast was attached to a hook on the ceiling of the cage, and the height of the hook was adjusted to an inclination of approximately 35° in a head‐down orientation. The rats were allowed to move freely around their cages using their front legs. Rats were checked daily for signs of tail lesions or discoloration.

### 2.3 Muscle preparation

At the end of the treatment, rats from both the CON and HU groups were deeply anesthetized with sodium pentobarbital. Once completely unresponsive to the stimulation, the plantaris muscles of rats were dissected and frozen in liquid nitrogen. Then, the rats were euthanized by exsanguination. Frozen plantaris muscles were homogenized in lysis buffer containing 40 mM Tris, 8 M urea, 4% CHAPS, 65 mM dithiothreitol, 1 mM ethylenediamine‐*N,N,N′*, *N′*‐tetraacetic acid, disodium salt, dihydrate, 20 mM N‐ethylmaleimide, and cOmplete™ Protease Inhibitor (11836170001; Roche Applied Science). The homogenates were then centrifuged at 15,000× g for 15 min at 4°C and the middle layer containing the proteins was carefully collected.

### 2.4 Western blot analysis

Equal amounts of protein (50 μg/lane) were loaded onto polyacrylamide gels, and the proteins were separated by sodium dodecyl sulfate‐polyacrylamide gel electrophoresis (SDS‐PAGE) using 8%, 10%, 12%, and 3%–10% gradient (u‐PAGEL H, UH‐T310, and ATTO) gels. Proteins in the gels were then transferred onto polyvinylidene fluoride membranes (IPVH00010; Immobilon‐P, Millipore‐Merk) using a mini trans‐blot cell (1703930JA; Bio‐Rad) at a constant voltage of 60 V for 2 h. Non‐specific binding sites were blocked for 1 h at room temperature with 5% bovine serum albumin (BSA) or 5% skim milk in Tris‐buffered saline containing Tween 20 (TBST), pH7.6. The membranes were incubated overnight at 4°C or for 1 h at room temperature with primary antibodies (Table 1), diluted in TBST containing 3% BSA, 3% skim milk, or Can Get Signal Immunoreaction Enhancer Solution 1 (NKB‐101T; TOYOBO Co., Ltd). Subsequently, the membranes were incubated for 1 h at room temperature with alkaline phosphatase (AP)‐conjugated secondary antibodies (Table 1), diluted in TBST containing 1% BSA, 1% skim milk, or Can Get Signal Solution 2. The signals were then visualized using Immunstar‐AP substrate (#1705018, Bio‐Rad), and the membranes were exposed to Hyperfilm ECL (28,906,836, Cytiva). Primary and secondary antibodies used in this study are listed in Table 1.

**TABLE 1:** List of antibodies used for Western blot analysis.

Antibody	Manufacturer	Catalog number	RRID	Dilution
GLUT1 (rabbit polyclonal)	Bioss	bs‐0472	AB_10856433	1:5000
Phospho‐HMGCR(Ser872) (rabbit polyclonal)		bs‐4063R	AB_10856788	1:5000
PDHA1 (phospho‐S293) (rabbit monoclonal)	Abcam	ab177461	AB_2756339	1:5000
Phospho‐ACLY(Ser455) (rabbit polyclonal)	Invitrogen	PA5‐97395	AB_2809197	1:4000
HMGCR (rabbit monoclonal)	Novus Biologicals	NBP3‐15641	AB_2943678	1:2000
Ras (mouse monoclonal)	BD Biosciences	610001	AB_397424	1:4000
GLUT4 (mouse monoclonal)	Proteintech	66846‐1‐Ig	AB_2882186	1:3000
PDHE1α (rabbit polyclonal)		18068‐1‐AP	AB_2162931	1:5000
IDH2 (rabbit polyclonal)		15932–1	AB_2264612	1:3000
IDH3A (rabbit polyclonal)		15909‐1‐AP	AB_2123282	1:2400
OGDH (rabbit polyclonal)		15212‐1‐AP	AB_2156759	1:10000
IDH1 (rabbit polyclonal)		12332‐1‐AP	AB_2123159	1:2000
ACLY (rabbit polyclonal)		15421‐1‐AP	AB_2223741	1:4000
MCU (rabbit monoclonal)	Cell Signaling Technology	#14997	AB_2721812	1:1000
CBARA1/MICU1 (rabbit monoclonal)		#12524	AB_2797943	1:1000
Rap1A/Rap1B (rabbit polyclonal)		#4938	AB_2177112	1:2000
Pan‐AKT (rabbit monoclonal)		#4691	AB_915783	1:1000
Phospho‐AKT (Ser473) (rabbit monoclonal)		#4060	AB_2315049	1:4000
Phospho‐AKT (Thr308) (rabbit monoclonal)		#13038	AB_2629447	1:2000
GSK‐3β (rabbit monoclonal)		#12456	AB_2636978	1:2000
Phospho‐GSK‐3β (Ser9) (rabbit monoclonal)		#5558	AB_10013750	1:2000
FOXO1 (rabbit monoclonal)		#2880	AB_2106495	1:2000
Phospho‐FoxO1 (Ser256) (rabbit polyclonal)		#9461	AB_329831	1:2000
P70S6K (rabbit monoclonal)		#34475	AB_2943679	1:2000
Phospho‐p70S6K(Thr389) (rabbit monoclonal)		#9234	AB_2269803	1:2000
4E‐BP1 (rabbit monoclonal)		#9644	AB_2097841	1:2000
Phospho‐4E‐BP1(Thr37/46) (rabbit monoclonal)		#2855	AB_560835	1:2000
IRS‐1 (rabbit monoclonal)		#3407	AB_2127860	1:2000
Phospho‐IRS‐1 (Ser307) (rabbit polyclonal)		#2381	AB_330342	1:2000
ERK1/2 (rabbit monoclonal)		#4695	AB_390779	1:2000
Phospho‐ERK1/2 (Thr202/Tyr204) (rabbit monoclonal)		#4370	AB_2315112	1:4000
Rheb (rabbit monoclonal)		#13879	AB_2721022	1:2000
VDAC1 (mouse monoclonal)	Santa Cruz Biotechnology	sc‐390996	AB_2750920	1:1000
AP‐conjugated anti‐mouse IgG	Jackson ImmunoResearch Laboratories	715‐055‐151	AB_2340778	1:100,000
AP‐conjugated anti‐rabbit IgG		711‐055‐152	AB_2340591	1:100,000

The total protein on the membrane was detected using SYPRO Ruby protein blot stain (S11791, Invitrogen; 50565, Lonza Rockland, Inc.). Membranes were stained with SYPRO Ruby Protein Blot Stain immediately after protein transfer onto the membranes. The signals were visualized using an LED Transilluminator (LED470‐TR60W; MeCan imaging), and images were acquired using a digital camera.

### 2.5 Image analysis

The film images were scanned for densitometric analysis. Western blot analysis and SYPRO Ruby staining signals across each lane were quantified using ImageJ software (National Institutes of Health, Bethesda, MD; RRID:SCR_003070).

### 2.6 Statistical analysis

Relative muscle weight was calculated by dividing the muscle weight by the body weight. We have previously reported the body weight of both groups, documenting a body weight reduction in the HU group after 2 weeks of HU (Uda et al., 2020). The protein expression levels were normalized to the total protein levels. Phosphorylation levels are expressed as the ratio of phosphorylated protein to the expression level of each unphosphorylated form of the protein. In addition, expression levels of mitochondrial proteins were normalized to the expression levels of voltage‐dependent anion‐selective channel protein 1, a mitochondrial loading control protein. Furthermore, the ratio of mitochondrial calcium uptake protein 1 (MICU1) to calcium uniporter protein (MCU) was calculated, and the gamma form of eukaryotic translation initiation factor 4E‐binding protein 1 (4E‐BP1) was normalized to total 4E‐BP1 expression. All data are presented as the means ± SD. Statistical analyses were performed using IBM SPSS Statistics software version 24.0 (RRID:SCR_002865). Normality was confirmed using the Shapiro–Wilk test. Based on the data distribution, either a two‐tailed independent samples *t*‐test or a two‐tailed Mann–Whitney *U*‐test was used to analyze differences between the CON and HU groups. Statistical significance was set at *p* < 0.05.

## 3 RESULTS

### 3.1 Plantaris muscle weight and relative muscle weight

**FIGURE 1:**
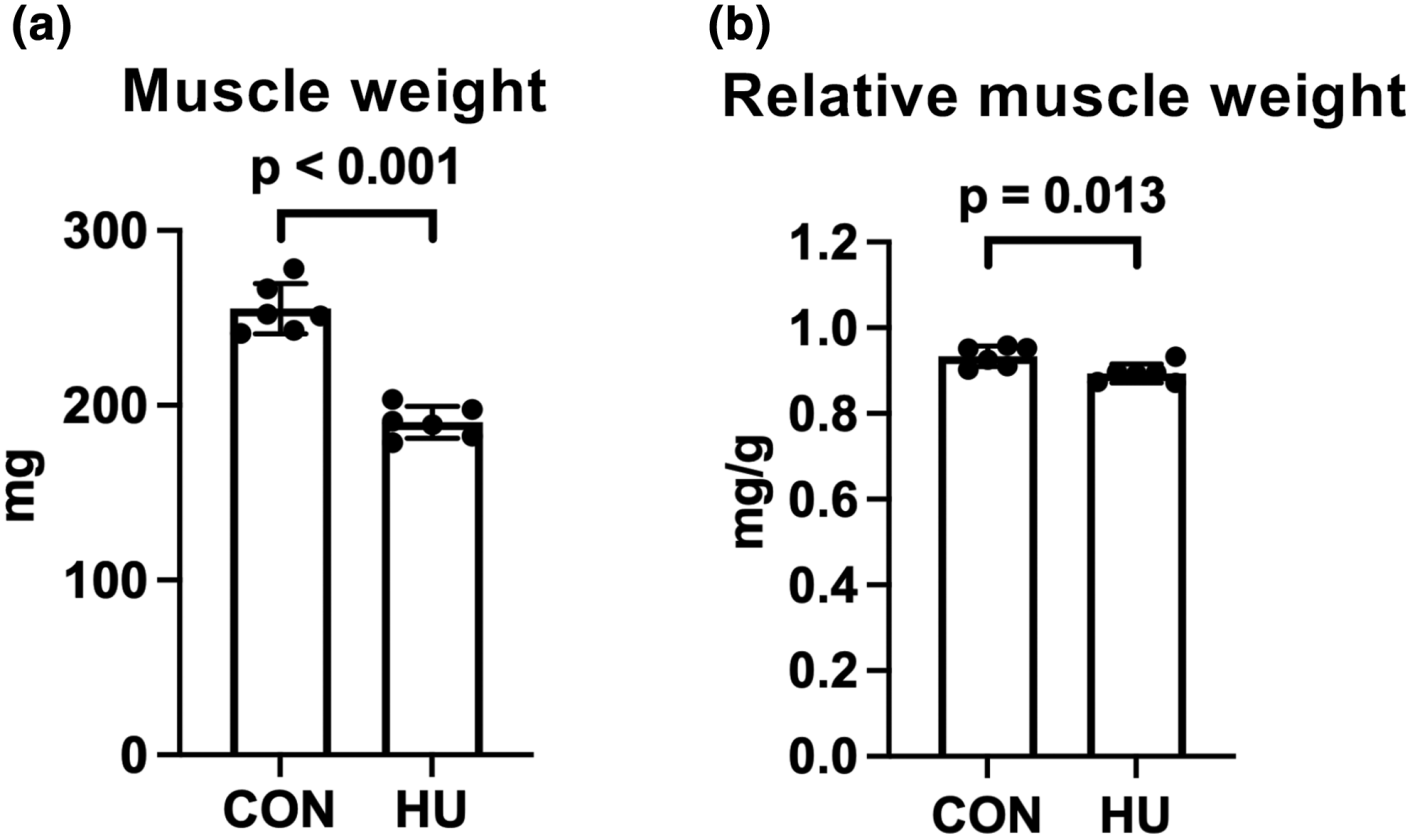
Effects of hindlimb unloading on the plantaris muscle weight and relative weight. Changes in plantaris muscle weight (a) and plantaris muscle weight relative to body weight (b). *n* = 6 for each group. CON, control; HU, hindlimb unloading. Data are expressed as mean ± standard deviation (SD). A two‐tailed independent samples *t*‐test was used to analyze the differences between the CON and HU groups.

Muscle weight and muscle weight relative to body weight in the HU group were significantly reduced by 25.5% (*p* < 0.001) and 4.3% (*p* = 0.013), respectively, compared with those in the CON group (Figure 1a,b).

### 3.2 Expression of glucose transporters, pyruvate dehydrogenase E1α subunit (PDHE1α), and proteins related to mitochondrial calcium uptake

**FIGURE 2:**
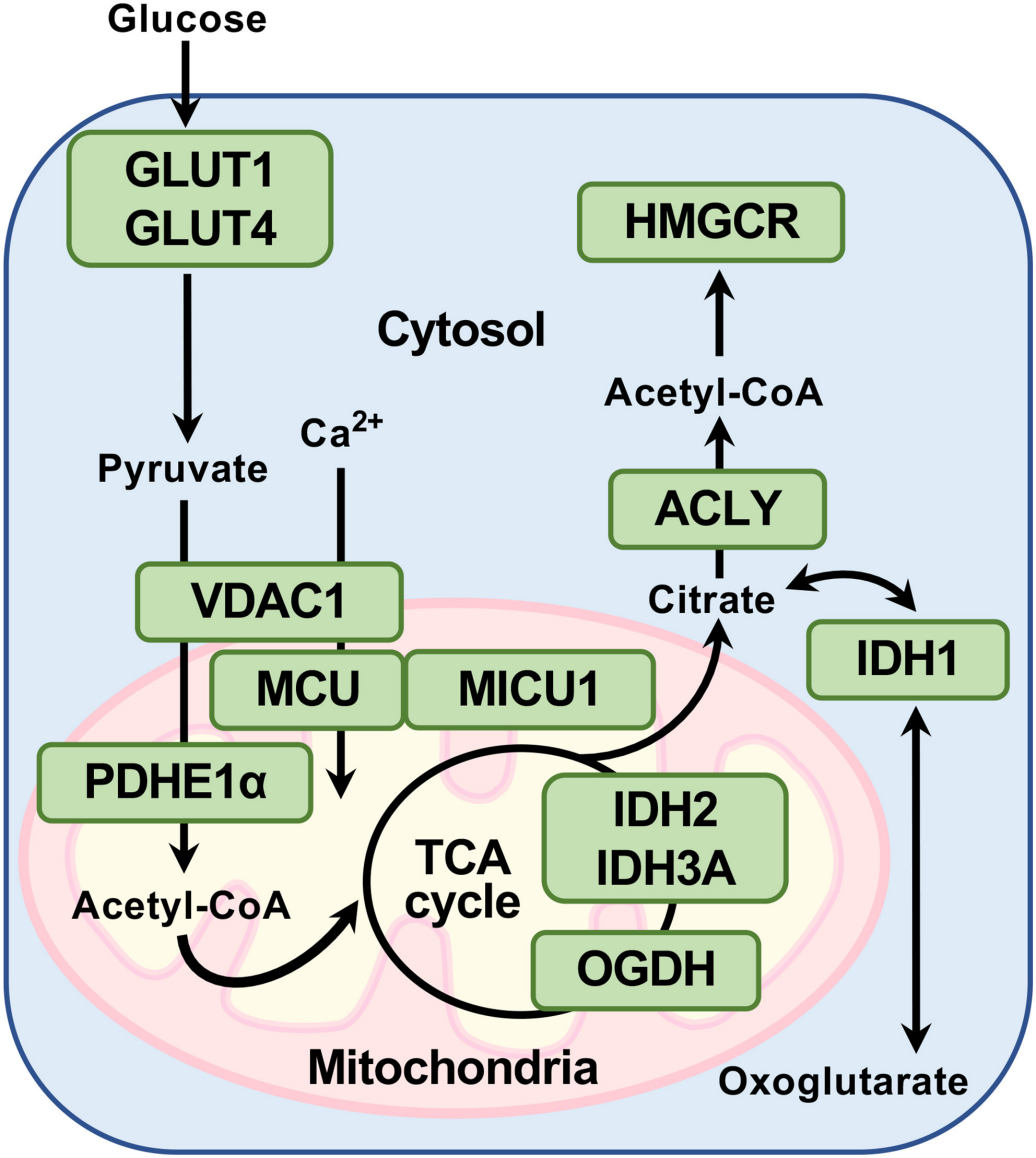
Enzymes and their related proteins involved in the metabolic processes evaluated in this study. Glucose transporter 1 (GLUT1) and GLUT4 facilitate the uptake of glucose into the cells. Pyruvate dehydrogenase E1 alpha (PDHE1α) forms a PDH complex that participates in pyruvate oxidation. PDHE1α is activated by dephosphorylation. Mitochondrial calcium uniporter (MCU) is a calcium channel protein in the inner mitochondrial membrane; mitochondrial calcium uptake 1 (MICU1) is the gatekeeper of the MCU channel. Voltage‐dependent anion‐selective channel protein 1 (VDAC1) is a mitochondrial outer membrane protein and participates in translocating substances such as pyruvate and calcium ions across the mitochondria. Isocitrate dehydrogenase 2 (NADP) (IDH2) and isocitrate dehydrogenase 3 (NAD) (IDH3A) catalyze the conversion of isocitrate to oxoglutarate (α‐ketoglutarate), which is subsequently converted to succinyl‐CoA by oxoglutarate dehydrogenase (OGDH). Cytosolic citrate is converted to acetyl‐CoA by ATP citrate lyase (ACLY) or to oxoglutarate via isocitrate by IDH1, which reversibly converts oxoglutarate to isocitrate. 3‐Hydroxy‐3‐methylglutaryl‐CoA reductase (HMGCR) converts cytosolic acetyl‐CoA to mevalonate in several steps.

**FIGURE 3:**
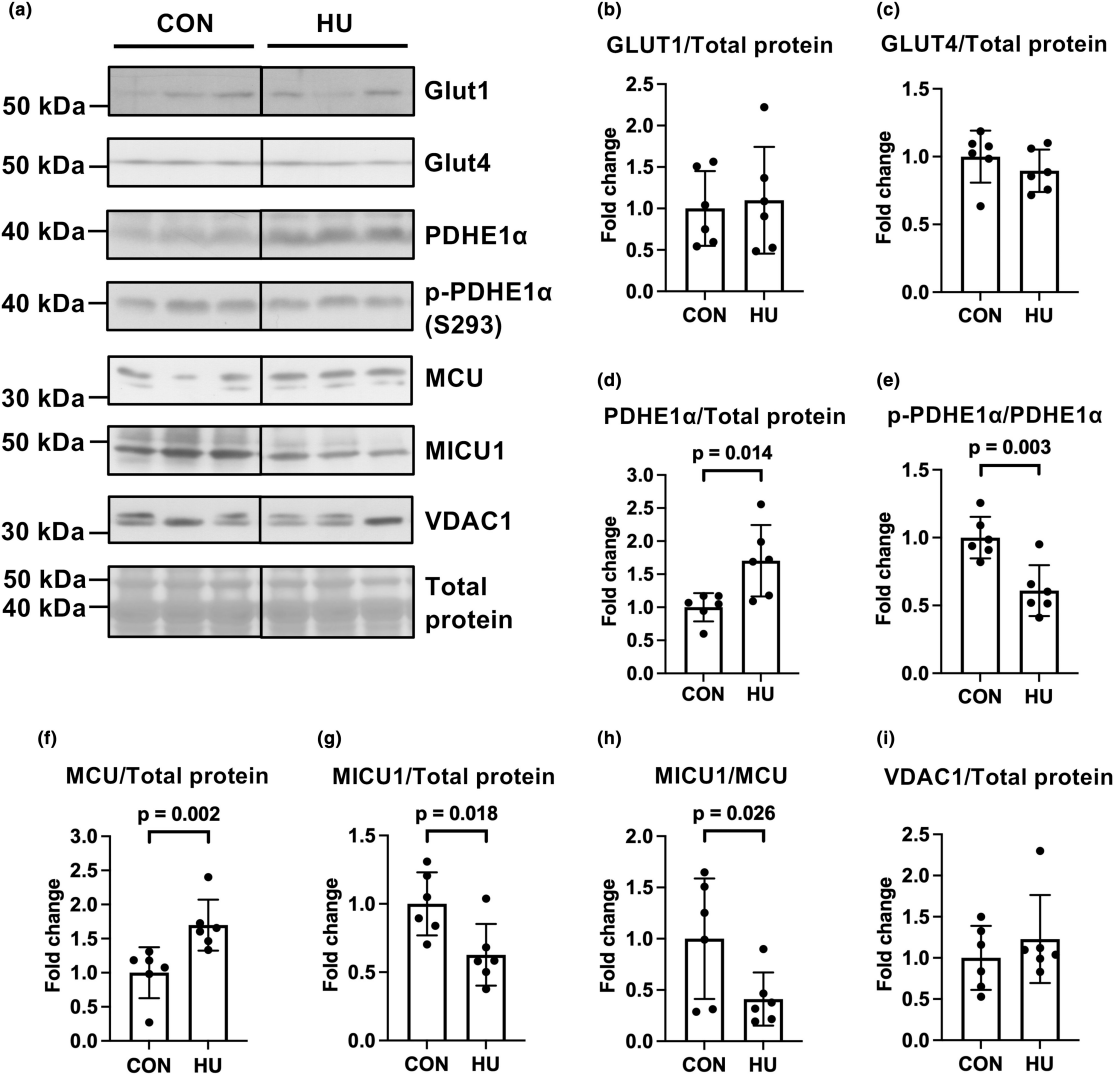
Effects of hindlimb unloading on the expression and phosphorylation levels of proteins related to glucose uptake, pyruvate oxidation, and calcium uptake in the mitochondria. Representative results of Western blotting showing glucose transporter 1 (GLUT1), GLUT4, pyruvate dehydrogenase E1 alpha (PDHE1α), phospho‐PDHE1α (p‐PDHE1α), mitochondrial calcium uniporter (MCU), mitochondrial calcium uptake 1 (MICU1), voltage‐dependent anion‐selective channel protein 1 (VDAC1), and total protein (a). Comparisons of GLUT1 (b), GLUT4 (c), PDHE1α (d), phospho‐PDHE1α (e), MCU (f), MICU1 (g), the ratio of MICU1 to MCU (h), and VDAC1 (i) expression levels between the control (CON) and hindlimb unloading (HU) groups. Data are expressed as mean ± standard deviation (SD). *n* = 6 for each group. Significant differences in MCU (f), the ratio of MICU1 to MCU (h), and VDAC1 (i) expression between the CON and HU groups were analyzed using a two‐tailed Mann–Whitney *U*‐test, other data were analyzed using a two‐tailed independent samples *t*‐test.

Figure 2 shows the enzymes and their related proteins involved in the metabolic processes evaluated in this study. We first examined the expression of glucose transporter (GLUT) 1, GLUT4, and pyruvate dehydrogenase (PDH) E1α. NO differences in GLUT1 (*p* = 0.765) or GLUT4 (*p* = 0.324) expressions were observed between the two groups (Figure 3a–c). In contrast, HU significantly elevated PDHE1α expression normalized to total protein (1.7‐fold vs. the CON group, *p* = 0.014; Figure 3a,d), but not normalized to VDAC1 expression (*p* = 0.127; Figure S1). In addition, HU significantly decreased phospho‐PDHE1α (S293) levels (0.6‐fold vs. the CON group, *p* = 0.003; Figure 3a,e). These results suggest that PDHE1α was activated in the plantaris muscles after 14 days of HU.

Given that decreased phospho‐PDHE1α (S293) levels are indirectly induced by increased mitochondrial calcium (Denton, 2009), we examined the expression of MCU and MICU1, which regulate mitochondrial calcium uptake (Mammucari et al., 2018). HU significantly increased MCU expression normalized to total protein (1.7‐fold vs. the CON group, Mann–Whitney *U*‐test, *p* = 0.002; Figure 3f) and VDAC1 expression (*p* = 0.049; Figure S1). In contrast, HU significantly downregulated MICU1 expression normalized to total protein (0.6‐fold vs. the CON group, *p* = 0.018; Figure 3a,g), but not normalized to VDAC1 expression (*p* = 0.093; Figure S1). The ratio of MICU1 to MCU, the reduction of which indicates increased calcium uptake by the mitochondria (Paillard et al., 2017), was significantly reduced in the HU group (0.4‐fold vs. the CON group, Mann–Whitney *U*‐test, *p* = 0.026; Figure 3h). HU did not alter the expression of VDAC1 normalized to total protein (Figure 3i). These results suggest that increased calcium uptake into the mitochondria may be responsible for PDHE1α activation in the HU group.

### 3.3 Expression of mitochondrial rate‐limiting enzymes and enzymes that convert cytosolic citrate to mevalonate

**FIGURE 4:**
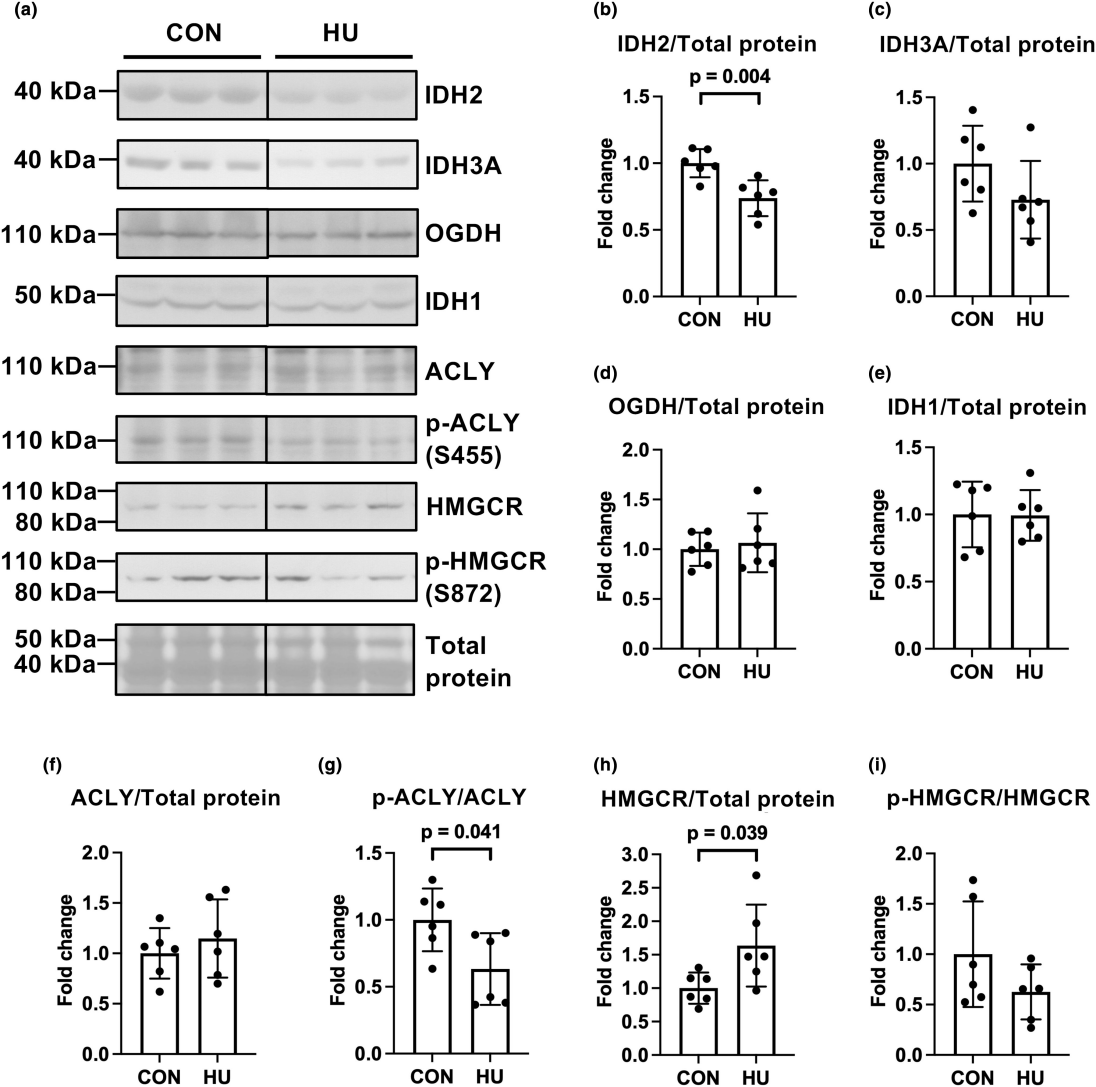
Effects of hindlimb unloading on the expression of metabolic enzymes in the mitochondria and cytosol. Representative results of Western blotting showing expression of isocitrate dehydrogenase 2 (NADP) (IDH2), isocitrate dehydrogenase 3 (NAD) (IDH3A), oxoglutarate dehydrogenase (OGDH), IDH1, ATP citrate lyase (ACLY), phospho‐ACLY (p‐ACLY), 3‐hydroxy‐3‐methylglutaryl‐CoA reductase (HMGCR), phospho‐HMGCR (p‐HMGCR), and total protein (a). Comparisons of IDH2 (b), IDH3A (c), OGDH (d), IDH1 (e), ACLY (f), phospho‐ACLY (g), HMGCR (h), and phospho‐HMGCR (i) expression levels between the control (CON) and hindlimb unloading (HU) groups. Data are expressed as mean ± standard deviation (SD). *n* = 6 for each group. Significant differences in phospho‐ACLY (g) expression between the CON and HU groups were analyzed using a two‐tailed Mann–Whitney *U*‐test, and other data were analyzed using a two‐tailed independent samples *t*‐test.

Next, we evaluated the expression of isocitrate dehydrogenase (IDH) 2, IDH3A, and oxoglutarate dehydrogenase (OGDH), all rate‐limiting enzymes in the TCA cycle. The plantaris muscle comprises fast‐twitch oxidative glycolytic and fast‐twitch glycolytic fibers expressing IDH2 and IDH3A (Armstrong & Phelps, 1984; Murgia et al., 2015; Schiaffino et al., 2015). Herein, we observed that HU significantly reduced IDH2 expression normalized to total protein (0.7‐fold vs. the CON group, *p* = 0.004; Figure 4a,b) and VDAC1 expression (0.6‐fold vs. the CON group, *p* = 0.020; Figure S1). In addition, HU tended to decrease IDH3A expression normalized to total protein (*p* = 0.134; Figure 4a,c) and VDAC1 expression (*p* = 0.138; Figure S1). However, HU did not alter OGDH expression normalized to total protein (*p* = 0.651; Figure 4a,d) and VDAC1 expression (*p* = 0.405; Figure S1).

ATP‐citrate lyase (ACLY) provides substrates from mitochondria and cytosolic IDH1 to the mevalonate pathway (Mullen et al., 2016; Pietrocola et al., 2015) (Figure 2). IDH1 (*p* = 0.956) and ACLY (*p* = 0.453) expression levels did not differ between the groups (Figure 4a,e,f). However because ACLY is activated by allosteric regulation or phosphorylation (Batchuluun et al., 2022), we further examined ACLY phosphorylation levels. HU significantly diminished phospho‐ACLY (S455) levels (0.6‐fold vs. the CON group, Mann–Whitney *U*‐test, *p* = 0.041; Figure 4a,g). In contrast, HU significantly upregulated HMGCR expression (1.6‐fold vs. the CON group, *p* = 0.039; Figure 4a,h). Nevertheless, although HMGCR is inactivated by phosphorylation via AMP‐activated protein kinase (Clarke & Hardie, 1990; Gillespie & Hardie, 1992), phospho‐HMGCR (S872) levels did not differ between the groups (*p* = 0.153; Figure 4a,i).

### 3.4 Expression and phosphorylation of AKT and its related proteins

**FIGURE 5:**
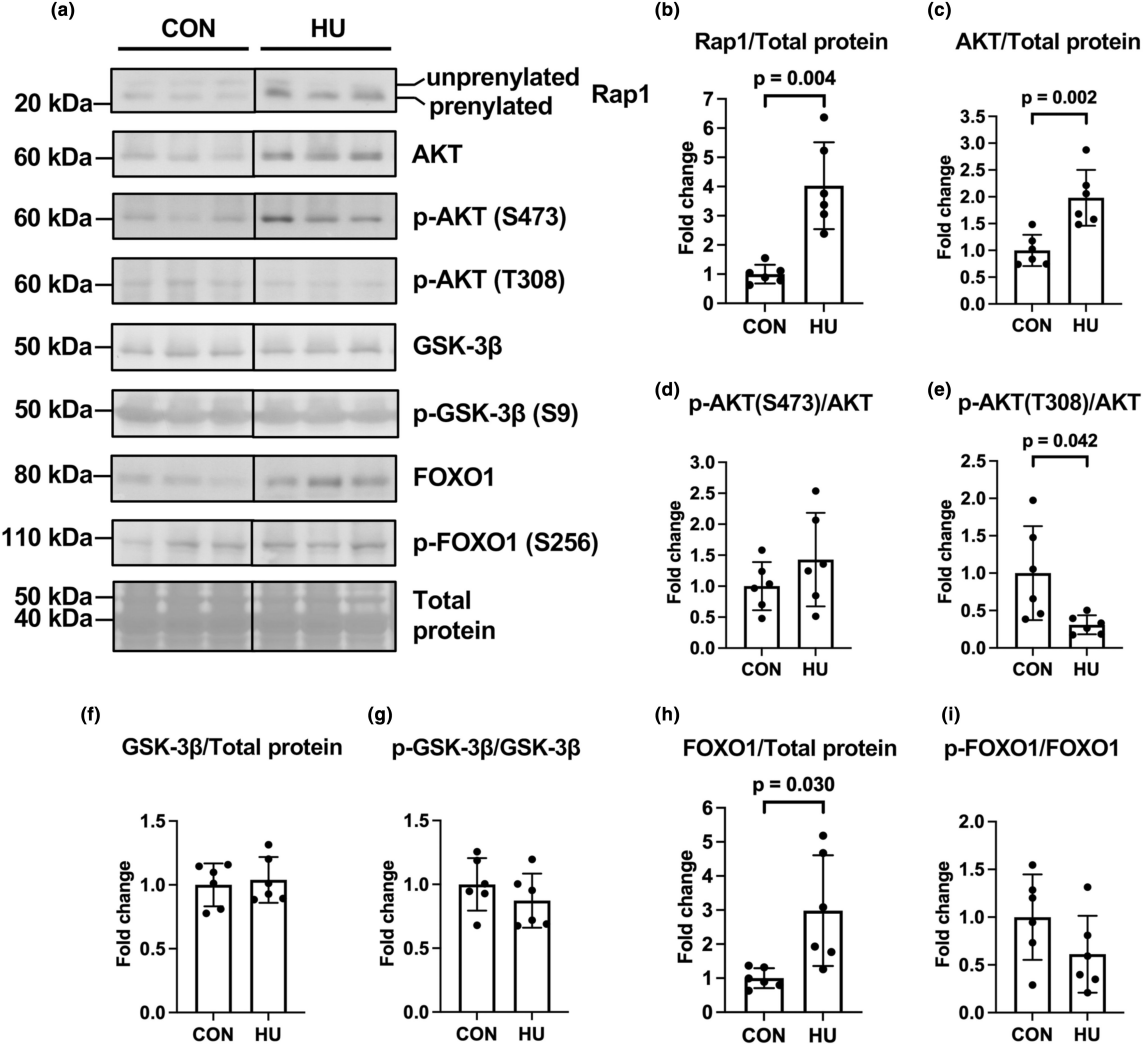
Effects of hindlimb unloading on the expression and phosphorylation of proteins in the AKT pathway and related proteins. Representative results of Western blotting showing expression of Ras‐related protein 1 (Rap1), AKT, phospho‐AKT (Ser473) (p‐AKT (S473)), phospho‐AKT (Thr308) (p‐AKT(T308)), glycogen synthase kinase‐3β (GSK‐3β), phospho‐GSK‐3β (p‐GSK‐3β) (S9), forkhead box protein O1 (FOXO1), phospho‐FOXO1 (p‐FOXO1) (S256), and total protein (a). Comparisons of Rap1 (b), AKT (c), phospho‐AKT (S473) (d), phospho‐AKT (T308) (e), GSK‐3β (f), phospho‐GSK‐3β (S9) (g), FOXO1 (h), and phospho‐FOXO1(S256) (i) expression between the control (CON) and hindlimb unloading (HU) groups. Data are expressed as mean ± standard deviation (SD). *n* = 6 for each group. Significant differences between the CON and HU groups in all data were analyzed using a two‐tailed independent samples *t*‐test.

The mevalonate pathway is involved in AKT phosphorylation at Ser473 via mTORC2 (Jaskiewicz et al., 2018; Sanvee et al., 2021). mTORC2 is activated by Ras‐related protein 1 (Rap1), a small GTPase activated by prenylation (Jaskiewicz et al., 2018; Sanvee et al., 2021). Prenylated small GTPases, including Rap1, Ras, and Rheb, can be distinguished by differences in their migration speed using SDS‐PAGE; prenylated small GTPases migrate more rapidly than their unprenylated forms (Ahearn et al., 2012). HU significantly increased prenylated Rap1 expression (4‐fold vs. the CON group, *p* = 0.004; Figure 5a,b). In addition, HU significantly elevated pan‐AKT expression (2‐fold vs. the CON group, *p* = 0.002) but did not alter phospho‐AKT (S473) levels (*p* = 0.245) (Figure 5a,c,d). In contrast, HU significantly downregulated phospho‐AKT (T308) levels (0.3‐fold vs. the CON group, *p* = 0.042; Figure 5a,e).

Glycogen synthase kinase‐3β (GSK‐3β) and forkhead box protein O1 (FOXO1) are target proteins of AKT signaling. Expression (*p* = 0.705) and phosphorylation levels (*p* = 0.315) of GSK‐3β did not differ between the groups (Figure 5a,f,g). Alternatively, HU significantly increased the expression of FOXO1 (3‐fold vs. the CON group, *p* = 0.030) but not phospho‐FOXO1 (S256) (*p* = 0.145) (Figure 5a,h,i). These findings indicate that 14 days of HU did not activate AKT in the plantaris muscles.

### 3.5 Expression and phosphorylation of proteins in the mTORC1 signaling pathway

**FIGURE 6:**
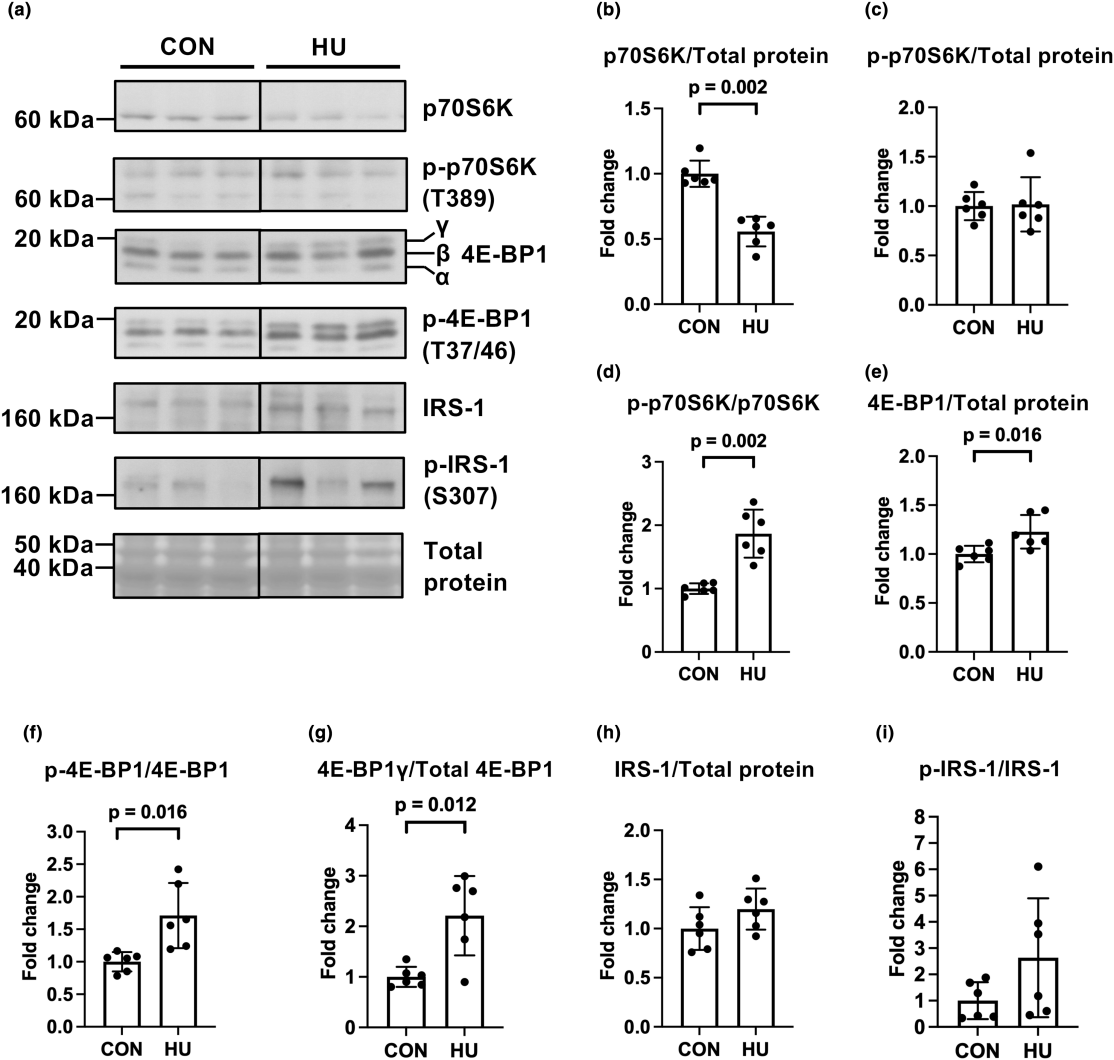
Effects of hindlimb unloading on the expression and phosphorylation of the mTORC1 signaling pathway. Representative results of Western blotting showing the expression of 70‐kDa ribosomal protein S6 kinase (p70S6K), phospho‐p70S6K (Thr389) (p‐p70S6K(T389)), 4E‐binding protein 1 (4E‐BP1), phospho‐4E‐BP1 (p‐4E‐BP1) (T37/46), insulin receptor substrate‐1 (IRS‐1), phospho‐IRS‐1 (Ser307) (p‐IRS‐1(S307)), and total protein (a). Comparisons of p70S6K (b), phospho‐p70S6K(T389)/total protein (c), phospho‐p70S6K(T389)/p70S6K (d), 4E‐BP1 (e), phospho‐4E‐BP1 (T37/46) (f), 4E‐BP1γ (g), IRS‐1 (h), and phospho‐IRS‐1(S307) (i) expression between the control (CON) and hindlimb unloading (HU) groups. Data are expressed as mean ± standard deviation (SD). *n* = 6 for each group. Significant differences between the CON and HU groups in p70S6K expression (b) were analyzed using a two‐tailed Mann–Whitney *U*‐test; other data were analyzed using a two‐tailed independent samples *t*‐test.

The 70‐kDa ribosomal protein S6 kinase (p70S6K) and 4E‐BP1 are downstream targets of the AKT/mTORC1 signaling pathway and are markers of mTORC1 signaling (Goodman, 2019). HU significantly diminished p70S6K expression (0.6‐fold vs. the CON group, Mann–Whitney *U*‐test, *p* = 0.002) but did not affect the ratio of phospho‐p70S6K (T389) to total protein (*p* = 0.892) (Figure 6a–c). Thus, HU significantly increased phosphorylation levels at T389 of p70S6K (1.9‐fold vs. the CON group, *p* = 0.002; Figure 6d). HU significantly increased 4E‐BP1 expression (1.2‐fold vs. the CON group, *p* = 0.016) and phosphorylation (Thr37/Thr46; T37/46) (1.7‐fold vs. the CON group, *p* = 0.016) (Figure 6a,e,f). Furthermore, HU significantly increased the expression of the inactive gamma form of 4E‐BP1 (2.2‐fold vs. the CON group, *p* = 0.012; Figure 6a,g). These results indicated that 14 days of HU activated the mTORC1 signaling pathway in the plantaris muscles.

Activation of the mTORC1 signaling pathway inhibits phospho‐AKT (T308) through inhibitory phosphorylation of insulin receptor substrate (IRS1)‐1 after denervation (Tang et al., 2014). Herein, the expression of IRS‐1 (*p* = 0.138) and phospho‐IRS‐1 (S307) (*p* = 0.142) did not differ between the groups (Figure 6a,h,i).

### 3.6 Expression and phosphorylation of non‐AKT proteins that activate mTORC1

**FIGURE 7:**
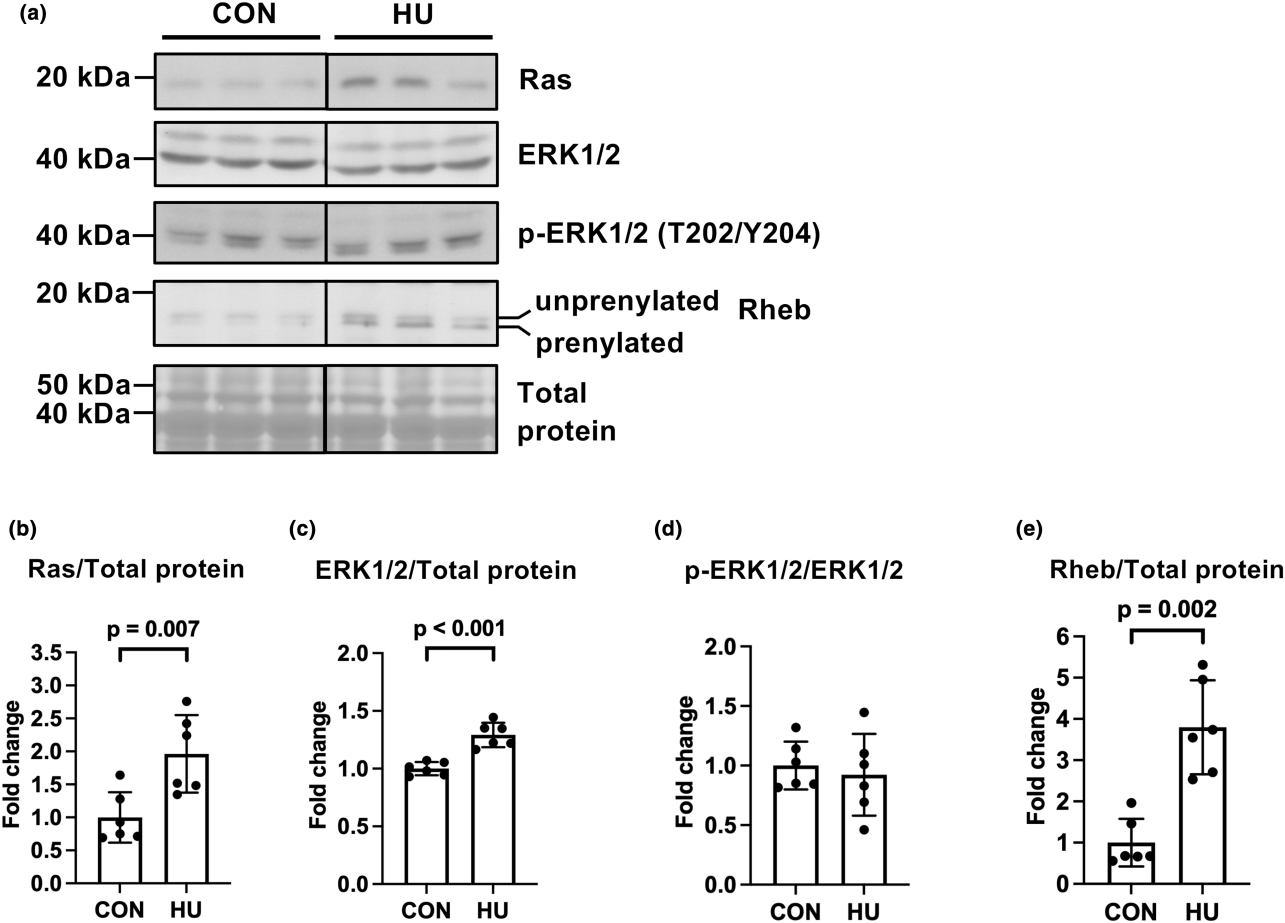
Effects of hindlimb unloading on the expression and phosphorylation of candidate proteins that activate the mTORC1 pathway. Representative results of Western blotting showing expression of Ras, extracellular signal‐regulated kinase 1/2 (ERK1/2), phospho‐ERK1/2 (Thr202/Tyr204) (p‐ERK1/2 (T202/Y204)), Ras homolog enriched in brain (Rheb), and total protein (a). Comparisons of Ras (b), ERK1/2 (c), phospho‐ERK1/2 (T202/Y204) (d), and Rheb (e) expression between the control (CON) and hindlimb unloading (HU) groups. Data are expressed as mean ± standard deviation (SD). *n* = 6 for each group. Significant differences between the CON and HU groups in Rheb expression (e) were analyzed using a two‐tailed Mann–Whitney *U*‐test; other data were analyzed using a two‐tailed independent samples *t*‐test.

Activation of the mevalonate pathway may induce the activation of the Ras/extracellular signal‐regulated kinase (ERK) and Rheb/mTORC1 signaling pathways (Basso et al., 2005; Castro et al., 2003; Lerner et al., 1995; Miyazaki et al., 2011). Therefore, we examined the protein expression levels of these signaling pathways. HU significantly increased expression levels of Ras (2‐fold vs. the CON group, *p* = 0.007) and ERK1/2 (1.2‐fold vs. the CON group, *p* < 0.001) (Figure 7a–c). In contrast, the phosphorylation levels of ERK1/2 (Thr202/Tyr204; T202/Y204) did not differ between groups (*p* = 0.645; Figure 7a,d). These results indicate that HU does not activate the Ras/ERK1/2 pathway. In contrast, HU significantly upregulated the expression of prenylated RHEB (3.8‐fold vs. the CON group, Mann–Whitney *U*‐test, *p* = 0.002; Figure 7a,e).

## 4 DISCUSSION

In the present study, we examined the effects of HU on the mevalonate pathway, along with its upstream metabolic pathway and the AKT/mTORC1 signaling pathway in a fast‐twitch muscle. We found that HU reduced the plantaris muscle weight and relative plantaris muscle weight when compared with those of the control group. The reduction in both plantaris muscle weights was less than the reduction in soleus muscles weight under HU conditions observed in our previous report (Uda et al., 2020), indicating that plantaris muscles are less susceptible to disuse atrophy than soleus muscles. In plantaris muscles, we found that 14 days of HU activated PDHE1α and downregulated IDH2 expression. Furthermore, 14 days of HU elevated HMGCR expression, activated mTORC1 signaling pathways without AKT activation, and increased the expression of prenylated Rheb. Our results provide new insights regarding the contribution of altered metabolic processes related to the mevalonate pathway toward the activation of the muscle protein synthesis signaling pathway in fast‐twitch muscles upon muscle disuse atrophy.

Our results revealed that 14 days of HU activated PDHE1α in the plantaris muscles, indicating that 14 days of HU may increase pyruvate oxidation. However, HU did not increase expression levels of GLUT1 or GLUT4. These results are consistent with those reported previously, showing that GLUT4 expression was not altered, but the activity of hexokinase, a glycolytic enzyme, was increased in slow‐ and fast‐twitch muscles after 14 days of HU in F344 rats and that PDH was activated in the denervated fast‐twitch muscles of mice (Odeh et al., 2020; Stump et al., 1997). Moreover, constitutive PDH activation induced by deletion of pyruvate dehydrogenase kinase 2 and 4, which inactivate PDH via phosphorylation, increased glucose oxidation in skeletal muscles (Rahimi et al., 2014). Therefore, 14 days of HU may increase glucose oxidation in the unloaded plantaris muscles. The present results also suggest that PDHE1α activation in the HU group may be induced by increased calcium uptake into the mitochondria. Reportedly, MCU expression is upregulated by increased cytosolic calcium, which stimulates PDH activity (Shanmughapriya et al., 2015). Additionally, MICU1 expression increases upon inhibition of the pyruvate transporter, suggesting that selection for mitochondrial substrate utilization can regulate MICU1 expression (Nemani et al., 2020). Therefore, alterations in calcium homeostasis in the plantaris muscles after unloading may underlie the increased MCU expression, PDH activation, substrate utilization, and decreased MICU1 expression observed in our study.

HU significantly reduced IDH2 expression and tended to decrease IDH3A expression in the plantaris muscles. These results are consistent with those of a previous study, in which denervation downregulated the expression of IDH2 and IDH3A in fast‐twitch fibers (Lang et al., 2018). HU also activated the mevalonate pathway in the plantaris muscles. In contrast, we found that HU did not alter IDH1 expression, suggesting that citrate and oxoglutarate production by IDH1 did not differ between groups. Therefore, these results suggest that the efflux of citrate from the mitochondria to the cytosol may increase and that citrate in the cytosol may be involved in enhancing the mevalonate pathway in the plantaris muscles after 14 days of HU. A previous study has shown that the inhibition of mitochondrial aconitase in myotubes located upstream of IDHs in the TCA cycle could increase citrate efflux from the mitochondria to the cytosol (Arnold et al., 2022). This observation supports our hypothesis. We also detected reduced levels of phospho‐ACLY (S455) in the HU group. Given that phosphorylation of ACLY at this site diminishes the sensitivity of allosteric regulation by metabolites of the glycolytic pathway (Batchuluun et al., 2022; Potapova et al., 2000), downregulation of phospho‐ACLY (S455) may enhance the allosteric activation of ACLY. Thus, cytosolic citrate likely flows toward the mevalonate pathway through allosterically activated ACLY rather than IDH1 in unloaded fast‐twitch muscles.

Prenylation and overexpression of Rheb activates the mTORC1 signaling pathway without AKT activation (Basso et al., 2005; Castro et al., 2003; Goodman et al., 2010; You et al., 2015). Reportedly, enhanced protein farnesylation, a prenylation induced downstream of the mevalonate pathway, was shown to enhance prenylated Rheb expression and activated the mTORC1 signaling pathway in mouse cardiomyocytes (Xu et al., 2015). In the present study, we observed an increase in HMGCR expression, indicating that 14 days of HU may activate the mevalonate pathway in the plantaris muscles. Furthermore, 14 days of HU resulted in the activation of the mTORC1 signaling pathways, along with elevated prenylated Rheb levels in the plantaris muscles. In contrast, HU did not activate AKT in the plantaris muscles. Therefore, our results suggest that increased levels of Rheb, which may be induced via the activated mevalonate pathway, may activate mTORC1 signaling independently of AKT activation in the plantaris muscle after 14 days of HU. In conjunction with our findings demonstrating that PDHE1α activation and IDH2 downregulation may be involved in the enhanced mevalonate pathway activation, this model suggests that the metabolic changes related to the mevalonate pathway observed in this study may contribute to the activation of the mTORC1 signaling pathway. This hypothesis is further supported by observations that IDH2 knockout mice exhibit cardiac hypertrophy and have a greater percentage of large skeletal muscle fibers than wild‐type mice (Ku et al., 2015; Pan et al., 2020).

We found that HU for 14 days activated the mTORC1 signaling pathway but reduced phospho‐AKT (T308) levels, suggesting that negative feedback loop of mTORC1 signaling may have suppressed AKT (T308) phosphorylation in the HU group. However, this does not necessarily indicate that HU reduced AKT activity, given that AKT (S473), GSK‐3β (S9), and FOXO1 (S256) phosphorylation levels did not differ between groups. Thus, our results suggest that AKT activity is maintained, at least in part, in the plantaris muscles after HU. Moreover, the activation of the mevalonate pathway and increased levels of prenylated Rap1 levels in the HU group may be responsible for maintaining AKT activity in the unloaded plantaris muscles of these rats.

Increased p70S6K phosphorylation can stimulates protein synthesis. We observed that HU significantly enhanced p70S6K phosphorylation levels at T389. This result suggests that 14 days of HU can activate the mTORC1/p70S6K signaling pathway. However, the ratio of phospho‐p70S6K (T389) to total protein did not differ between groups. Therefore, the stimulation of protein synthesis by the mTORC1/p70S6K signaling pathway was potentially maintained at the control level but not increased in the plantaris muscles of the HU group despite the activation of the mTORC1/p70S6K signaling pathway.

Notably, p70S6K also participates in the negative feedback loop of mTORC1 (Um et al., 2004). We observed that p70S6K expression was reduced in the HU group. A similar phenomenon was observed in fast‐twitch muscles after 3 weeks of hindlimb immobilization (MacDonald et al., 2014), but not after 1 week (You et al., 2015), suggesting that prolonged HU and hindlimb immobilization could reduce p70S6K expression in the unloaded muscles. p70S6K‐deficient mice suppress the negative feedback from p70S6K to IRS‐1 (Um et al., 2004). Therefore, the significant decrease in p70S6K expression, as observed in the present study, following HU for 14 days may suppress the negative feedback of mTORC1 signaling and impact the degree of IRS‐1 (S307) phosphorylation in unloaded plantaris muscles. In contrast, we observed a reduction in phospho‐AKT (T308) levels, which could be attributed to the negative feedback of mTORC1 signaling in the HU group. The discrepancy between the suppression of negative feedback and the inhibition of phospho‐AKT (T308) suggests that other pathways may participate in inhibiting phospho‐AKT (T308). In this context, mTORC1 has been found to phosphorylate other serine residues and inhibit IRS‐1 (Khamzina et al., 2005; Tzatsos & Kandror, 2006; Yoneyama et al., 2018). Alternatively, a time lag may exist between the two phenomena, implying that the complete recovery of phospho‐AKT (T308) may require additional time.

This study used the plantaris muscle and HU as a muscle disuse model. Therefore, whether the phenomena observed in the present study occur in other muscles or muscle disuse models remains to be determined. Because we did not measure protein synthesis or the expression of enzymes involved in protein degradation, alterations in these processes remain elusive. Furthermore, we did not administer statins to the HU group because of the difficulty in undertaking new animal experiments, owing to the considerable time required for available experimental facilities, procedures for using the experimental facilities, animal care, and cost restrictions. Statin treatment under muscle disuse may generate further evidence regarding the contribution of the mevalonate pathway to mTORC1 activation and muscle atrophy. Metabolomic analysis of fast‐twitch muscles following muscle disuse would also provide further, comprehensive evidence of the effects of muscle disuse‐induced metabolic changes on the mTORC1 signaling pathway.

## 5 CONCLUSION

In conclusion, 14 days of HU activated PDHE1α, potentially via altered calcium homeostasis in the unloaded plantaris muscles. Fourteen days of HU may also increase citrate efflux from the mitochondria to the cytosol in these muscles. In addition, 14 days of HU increased HMGCR expression, activated mTORC1 signaling pathways without AKT activation, and upregulated prenylated Rheb levels in the unloaded plantaris muscles. These findings suggest that substrate supply to the mevalonate pathway may be increased, and the potentially activated mevalonate pathway may be involved in the activation of the Rheb/mTORC1 signaling pathway in fast‐twitch muscles under prolonged disuse conditions. These molecular mechanisms may underlie the low susceptibility of fast‐twitch muscles to disuse atrophy. Using appropriate experimental designs, future studies should examine whether the phenomena observed in the current study occur in other muscles and experimental models.

## AUTHOR CONTRIBUTIONS

Munehiro Uda conceived and designed the research, performed experiments, analyzed data, interpreted the results of experiments, prepared figures, drafted the manuscript, edited and revised the manuscript, and approved the final version of the manuscript. Toshinori Yoshihara and Noriko Ichinoseki‐Sekine performed experiments, interpreted the results of experiments, edited and revised the manuscript, and approved the final version of the manuscript. Takeshi Baba designed research, interpreted the results of experiments, edited and revised the manuscript, and approved the final version of the manuscript.

## FUNDING INFORMATION

This work was supported by JSPS KAKENHI grant numbers JP22K11557, JP19K11554, JP26560402 to MU.

### CONFLICT OF INTEREST STATEMENT

The authors declare that they have no conflicts of interest.

## 6 ETHICS STATEMENT

All experiments were approved by the Institutional Animal Care and Use Committee of Juntendo University (approval number: H27‐05) and were conducted according to the guiding principles for the Care and Use of Laboratory Animals set by the Physiological Society of Japan.

## Supporting information


Figure S1.

## Data Availability

All the relevant data have been included in the manuscript and Figure S1.
