# Supplementary material for: Effects of hindlimb unloading on the mevalonate and mechanistic target of rapamycin complex 1 signaling pathways in a fast‐twitch muscle in rats
Source: Physiol Rep. 2024 Mar 7;12(5):e15969. doi: 10.14814/phy2.15969 (PMC10920058; doi:10.14814/phy2.15969)
Supplement: Supplementary file 1 — Figure S1. [file PHY2-12-e15969-s001.pdf]

Supplementary Figure 1

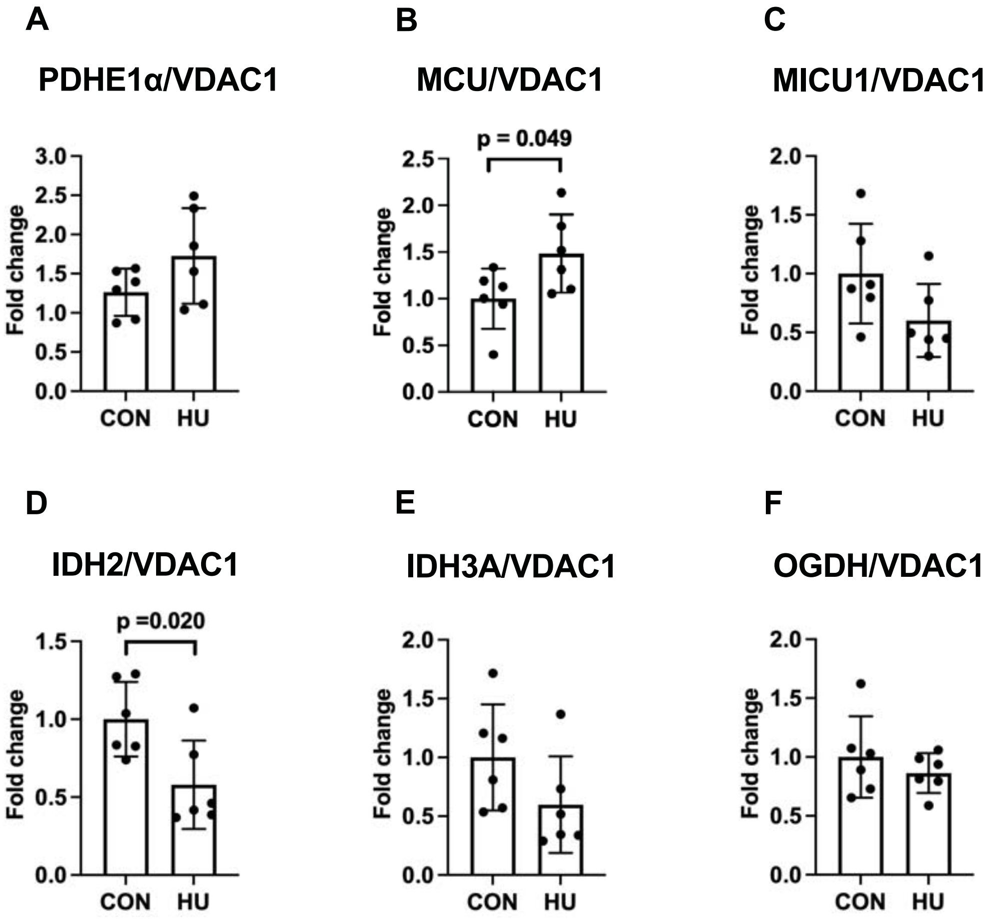

Supplementary Fig. 1. Comparisons of pyruvate dehydrogenase E1 alpha (PDHE1α) (A), mitochondrial calcium uniporter (MCU) (B), mitochondrial calcium uptake 1 (MICU1) (C), isocitrate dehydrogenase 2 (NADP) (IDH2) (D), isocitrate dehydrogenase 3 (NAD) (IDH3A) (E), and oxoglutarate dehydrogenase (OGDH) (F) expression between the control (CON) and hindlimb unloading (HU) groups. Data are expressed as mean ± standard deviation (SD). n = 6 for each group. Significant differences between the CON and HU groups in all data were analyzed using a two-tailed independent samples t-test.
